# Genetic association-based functional analysis detects *HOGA1* as a potential gene involved in fat accumulation

**DOI:** 10.3389/fgene.2022.951025

**Published:** 2022-08-12

**Authors:** Myungsuk Kim, Kye Won Park, Yeongseon Ahn, Eun Bi Lim, Soo Heon Kwak, Ahmad Randy, No Joon Song, Kyong Soo Park, Chu Won Nho, Yoon Shin Cho

**Affiliations:** ^1^ Natural Product Research Center, Korea Institute of Science and Technology, Gangneung, South Korea; ^2^ Department of Food Science and Biotechnology, Sungkyunkwan University, Suwon, South Korea; ^3^ Department of Biomedical Science, Hallym University, Chuncheon, South Korea; ^4^ Department of Internal Medicine, Seoul National University College of Medicine, Seoul, South Korea; ^5^ Smart Farm Research Center, Korea Institute of Science and Technology, Gangneung, South Korea

**Keywords:** exome sequencing, obesity, association analysis, adipogenesis, HOGA1, 3T3-L1, functional study

## Abstract

Although there are a number of discoveries from genome-wide association studies (GWAS) for obesity, it has not been successful in linking GWAS results to biology. We sought to discover causal genes for obesity by conducting functional studies on genes detected from genetic association analysis. Gene-based association analysis of 917 individual exome sequences showed that *HOGA1* attains exome-wide significance (*p*-value < 2.7 × 10^–6^) for body mass index (BMI). The mRNA expression of *HOGA1* is significantly increased in human adipose tissues from obese individuals in the Genotype-Tissue Expression (GTEx) dataset, which supports the genetic association of *HOGA1* with BMI. Functional analyses employing cell- and animal model-based approaches were performed to gain insights into the functional relevance of *Hoga1* in obesity. Adipogenesis was retarded when *Hoga1* was knocked down by siRNA treatment in a mouse 3T3-L1 cell line and a similar inhibitory effect was confirmed in mice with down-regulated *Hoga1*. *Hoga1* antisense oligonucleotide (ASO) treatment reduced body weight, blood lipid level, blood glucose, and adipocyte size in high-fat diet-induced mice. In addition, several lipogenic genes including *Srebf1*, *Scd1*, *Lp1*, and *Acaca* were down-regulated, while lipolytic genes *Cpt1l*, *Ppara*, and *Ucp1* were up-regulated. Taken together, *HOGA1* is a potential causal gene for obesity as it plays a role in excess body fat development.

## Introduction

Obesity, characterized by increased adipose tissue mass, poses a major unmet public health problem since it is a risk factor for many metabolic diseases and cancer (Kopelman, 2000; Ghaben and Scherer, 2019). Excess energy intake relative to energy expenditure during obesity promotes body weight gain by storing the remaining calories in triglycerides in the adipose tissue (Ng et al., 2014). The hypothalamus-adipose axis plays an important role in weight gain by regulating food intake and energy expenditure (Arora and Anubhuti, 2006). Obesity-related genes are known to be involved in appetite-related signals, adipocyte growth and differentiation, energy expenditure regulation, or insulin metabolism and adipose tissue inflammation (Gonzalez Jimenez, 2011). Considering the increasing incidence of obesity worldwide, discovering genetic factors is of fundamental significance to overcome the risk of obesity and its comorbidities. Although a number of obesity genetic factors were identified from numerous genetic studies, the molecular mechanism of adipogenesis has not been fully elucidated.

Obesity is a heritable disease and more than 250 common genetic variants are identified, mainly by genome-wide association studies (GWASs) for body mass index (BMI) over the past decade (Wen et al., 2012; Monda et al., 2013; Wen et al., 2014; Locke et al., 2015; Winkler et al., 2015; Akiyama et al., 2017). However, these GWAS loci (mostly common variants) do not fully explain BMI heritability (Manolio et al., 2009; Nolte et al., 2017). Moreover, most reported GWAS genes for BMI are not necessarily causal. Recently, a large-scale GWA meta-analysis combining 718,734 individuals discovered 14 rare or low-frequency genetic variants (minor allele frequency; MAF <5%) in 13 genes associated with BMI (Turcot et al., 2018). However, there are few examples that GWAS discoveries are linked to biology (Visscher et al., 2017).

Recently, deep sequencing of all known exons through high-throughput sequencing technology detected low-frequency disease-causing variants (Buermans and den Dunnen, 2014). This technology was employed in numerous genetic studies for diverse traits to better understand genetic heritability and to discover functional variants or genes for traits of interest (Yohe and Thyagarajan, 2017). In this regard, exome sequencing of 917 adult subjects was conducted to identify novel causal genes for obesity. Furthermore, biological approaches were employed to validate the potential causal genes detected from genetic association analysis.

## Materials and methods

### Subjects

A total of 917 subjects from the Korean population were recruited between 2001 and 2014 at Seoul National University Hospital (Kwak et al., 2018). The study protocol was approved by the institutional review board (IRB) of the Biomedical Research Institute at Seoul National University Hospital (IRB No. 1205-130-411), and written informed consent was obtained from each participant. All clinical investigations were conducted in accordance with the Declaration of Helsinki.

### Exome sequencing

Whole exome sequencing of 917 subjects was conducted as previously described (Kwak et al., 2017; Kwak et al., 2018). In brief, exome capture of genomic DNA was carried out using Agilent SureSelect v4+UTR and sequencing was conducted using an Illumina Hiseq (2000) sequencing system. The minimum and average read depths of coverage for the target region were 80X and 101X, respectively. The resulting sequence reads were aligned to the human reference genome buide 37 (GRCh37) and processed using Burrow-Wheeler Aligner (BWA) (Li and Durbin, 2009), Picard (http://broadinstitute.github.io/picard/), and Genome Analysis Toolkit (GATK) software (McKenna et al., 2010). Variant calling was performed using multi-sample HaplotypeCaller of GATK (McKenna et al., 2010). To ensure high-quality genotypes, variants were only called at sites where the low depth was ≥7, the genotype quality was ≥20, and the call rate was >0.90. Detailed quality control procedures for sequence data and the statistical summary of sequence read output were previously reported (Kwak et al., 2017).

### Association analysis

Of the total of 635,881 SNVs called from whole exome sequencing of 917 subjects, SNVs with a Hardy-Weinberg equilibrium test *p*-value < 10^–6^ were excluded from the subsequent analyses (Lim et al., 2021). The SKAT-O test implemented in the EPACTS software pipeline (http://genome.sph.umich.edu/wiki/EPACTS) (Kang et al., 2010) was employed for gene-based association analysis for BMI. Association analysis was adjusted for age, sex, and type 2 diabetes (T2D) status of subjects assuming an additive genetic model. In this study, the exome-wide significance threshold was determined at *p*-value < 2.7 × 10^–6^ based on a Bonferroni correction for a total of 18,379 loci reported in gene-based association analysis.

### Statistical analyses for quantitative data from 3T3-L1 cells and animal studies

In 3T3-L1 cell studies, relative gene expression levels measured from three independent experiments are presented as the mean ± standard deviation (SD). Group differences were assessed by the Wilcoxon rank-sum test. Statistical analyses were performed using R software. *P*-values < 0.05 were considered statistically significant.

Results of animal studies from more than three independent experiments are presented as the mean ± standard deviation (SD). Group differences were assessed using one-way analysis of variance (ANOVA), followed by Scheffe’s test. *P*-values < 0.05 were considered statistically significant. Statistical analyses were performed using SPSS 12.0 (SPSS Inc, Chicago, IL, United States).

### *HOGA1* expression in human tissues from the GTEx dataset

*HOGA1* expression data and phenotype information (phs000424.GTEx.v7. p2. c1. GRU) were obtained from the GTEx data portal (https://gtexportal.org/home/) (GTEx_Consortium, 2017). *HOGA1* expression was compared between 261 normal and 236 obese individuals by the Wilcoxon rank-sum test using RNA sequencing data of 848 adipocyte samples. BMIs above 30, between 25 and 30, and between 18.5 and 24.9 were considered obese, overweight, and normal according to the World Health Organization (WHO) obesity classification system. The association between BMI and *HOGA1* expression in a total of 848 adipocyte samples was tested by linear regression analysis adjusted for age, sex, and race.

*HOGA1* expression was compared between 49 normal and 49 obese individuals using RNA sequencing data of 188 liver tissue samples from the GTEx dataset. Group differences were assessed by the Wilcoxon rank-sum test. Statistical analyses were performed using R software. *P*-values < 0.05 were considered statistically significant.

### Mouse 3T3-L1 cell culture and Oil-Red-O staining

3T3-L1 preadipocytes derived from mouse 3T3 cells were grown in DMEM (Dulbecco’s modified Eagle’s medium) culture medium supplemented with 10% bovine calf serum (BCS) and 1% penicillin in a humidified incubator containing 5% CO_2_ at 37°C. After the 3T3-L1 preadipocytes became 100% confluent, the medium was replaced with a differentiation medium containing 10% FBS (fetal bovine serum), 0.5 mM isobutylmethylxanthine, 1 μM dexamethasone, and 1 μg/ml insulin and cultured for 2 days. The medium was replaced with a differentiation medium every 2 days. On day 7, cells were washed twice with phosphate-buffered saline, fixed with 10% formalin for 20 min at room temperature, and then stained with 0.5% Oil-Red-O in isopropanol for 20 min. Cells were washed with water and images of each dish were taken using an Eclipse TE 2000U inverted microscope with twin CCD cameras (Nikon, Tokyo, Japan).

### Transfection of 3T3-L1 cells with siRNA

*Hoga1* siRNAs and scrambled control siRNA were designed according to GenBank accessions (Supplementary Table S2). Two days before incubating in the differentiation medium, 3T3-L1 cells were transfected with control or siRNA specific to *Hoga1* (100 pmol). siRNA was incubated with Lipofectamine RNAiMAX (Invitrogen, United States) in DMEM for 20 min at room temperature before transfection. Six hours after transfection, the medium was replaced with a culture medium and further induced into adipocytes as described above. Differentiated adipocytes were assessed by Oil-Red-O staining and analyzed by quantitative real-time polymerase chain reaction (qRT-PCR) of adipogenic markers.

### Animal experiments

Twenty one 5 week-old male C57BL/6J mice (Central Laboratory Animal Inc, Seoul, Korea) were housed in a controlled environment (25 ± 2^○^C, 55 ± 5% relative humidity, 12 h light–dark cycle). The mice were allowed free access to food and tap water during the experiment. To study the role of *Hoga1 in vivo*, antisense oligonucleotide (ASO) were used as previously described (Song et al., 2016). Briefly, two independent siRNAs for *Hoga1* were designed for the mouse *Hoga1* mRNA sequence and screened to select the most potent ASO (Supplementary Table S2). A control ASO with no complementary binding to any known gene sequence was synthesized (Genolution, Seoul, Korea) and diluted in a buffer containing 1 mmol/L ethylenediaminetetraacetic acid (EDTA) and 10 mmol/L TrisHCl, pH 7.4. After acclimatization for 2 weeks, mice were randomly divided into two groups: NCD (N = 7) and HFD (N = 14). The HFD group was fed a rodent diet (D12451, Research Diets, New Brunswick, NJ, United States) wherein 60% of the energy came from fat, 20% from carbohydrates, and 20% from protein. The fourteen mice in the HFD group were equally divided into two groups of seven and intraperitoneally injected with validated *Hoga1* ASO (HFD Hoga1 ASO group), or control ASO (HFD vehicle group) twice per week (25 mg/kg) for 9 weeks. The NCD vehicle group was intraperitoneally injected with control ASO twice per week (25 mg/kg) for 9 weeks. The body weight and food intake were measured every week during animal experiments. All mice were sacrificed by cervical dislocation after overnight fasting at the end of *Hoga1* ASO treatment. Fat pads and livers were removed, weighed, and frozen in liquid nitrogen. This study adhered to the Guide for the Care and Use of Laboratory Animals developed by the Institute of Laboratory Animal Resources of the National Research Council. The study protocol was approved by the Institutional Animal Care and Use Committee of the Korea Institute of Science and Technology in Seoul, Korea.

### Histological and biochemical analyses

Blood samples were collected by cardiac puncture from all mice, and serum was separated by centrifugation at 7,000 rpm for 10 min and stored at −70^○^C for the subsequent analysis. White adipose tissue (WAT) and liver obtained from all mice were embedded in tissue-freezing medium (Leica, Wetzlar, Germany). Histological sections (5 μm thickness) of the tissues were cut, deparaffinized in xylene, and rehydrated in an alcohol solution gradient. After fixation, staining with hematoxylin and eosin (H&E) was performed, and adipocyte size was analyzed with an Eclipse TE 2000U inverted microscope with twin CCD cameras (×100 magnification; Nikon, Tokyo, Japan). TG, TC, and glucose levels were measured enzymatically using commercial kits (Asan Pham, Seoul, South Korea). Serum leptin, MCP-1, TNF-α, IL-6, and insulin were quantified using enzyme-linked immunosorbent assay (ELISA) kits (Abcam, Cambridge, United Kingdom). HOMA-IR was calculated using glucose and insulin levels. Each commercial kit was used according to the manufacturer’s instructions.

### Western blot

Epididymal WAT and liver specimens from all mice (approximately 20 mg each) were homogenized in lysis buffer supplemented with a protease inhibitor cocktail (Sigma-Aldrich, Saint Lousis, MO, United States). Western blotting was performed as described in a previously published study (Kim et al., 2017). Target proteins were detected using specific antibodies with enhanced chemiluminescence (ECL) detection system (Amersham Pharmacia Biotech, Piscataway, NJ, United States) and visualized with a LuminoImager (LAS-3000 Bio Imaging Analysis System; Fuji Film Co., Tokyo, Japan).

### Quantitative reverse transcriptase-polymerase chain reaction (qRT-PCR)

Total RNA was isolated from 3T3-L1 cells, livers, or WAT using Trizol reagent (Invitrogen, Carlsbad, CA, United States). Total RNA was quantified by nanodrop (Invitrogen, United States). cDNA was synthesized from 1 µg aliquots of the total RNA in a reaction mixture containing oligo (dT) and reverse transcription premix (ELPIS-Biotech, Daejeon, Korea). qRT-PCR was performed as described previously (Kim et al., 2017) using the SYBR Green master mix (Roche, Indianapolis, IN, United States) in the Light Cycler 480 real-time PCR system (Roche, Indianapolis, IN, United States). mRNA expression levels encoded by specific mouse genes were quantified using the specific primers listed in Supplementary Table S3.

### Reagents and materials

3T3-L1 cells were purchased from the American Type Culture Collection (Manassas, VA, United States). Oil-Red-O, 3-(4,5-dimethylthiazol-2-yl)-2,5-diphenyltetrazolium bromide (MTT), and other reagents were purchased from Sigma-Aldrich (St. Louis, MO, United States). FBS, penicillin/streptomycin and DMEM were purchased from Hyclone (Logan, UT, United States). BCS was purchased from Gibco (Grand Island, NY, United States). Antibodies against SREBP-1c, SCD1, FAS, PPARα, PGC-1α, CPT-1L, and β-actin, and HRP-linked anti-rabbit and anti-mouse IgG secondary antibodies were from Cell Signaling Technology (Beverly, MA, United States) and Santa Cruz Biotechnology (Santa Cruz, CA, United States), while the antibody against Hoga1 was from Abcam (Cambridge, United Kingdom).

## Results

### Genetic association analysis for BMI

Genetic association analysis using whole exome sequencing (WES) data from 917 subjects was performed to identify the causal genes for obesity (Kwak et al., 2018). Among approximately 674,000 single nucleotide variants (SNVs) in WES, common variants (MAF ≥5%), low frequency variants (0.5% ≤ MAF <5%), and rare variants (MAF <0.5%) accounted for 16, 11, and 73%, respectively. Moreover, exonic variants accounted for approximately 34%.

**FIGURE 1:**
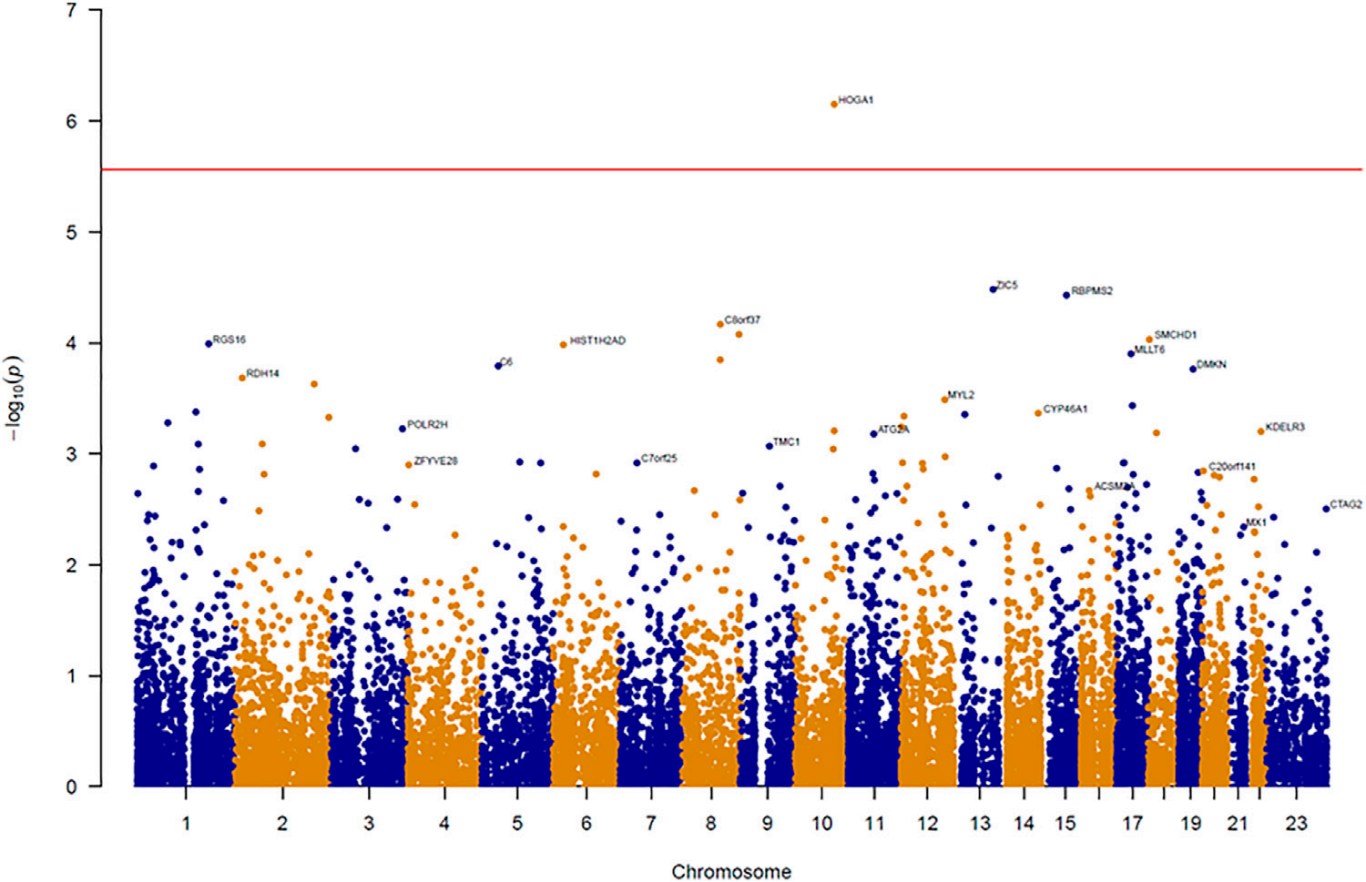
Manhattan plot of results from gene-based association analysis for BMI. The negative logarithm of the association *p*-value for genes distributed in the autosomal genome is represented as a dot. The red line represents the exome-wide significant *p*-value (*p*-value < 2.7 × 10^–6^).

An association test at the gene level was applied by grouping together rare variants to overcome the low power of rare variant association analysis. The SKAT-O test for gene-based analysis was performed to detect genes associated with BMI across all exonic loci (Figure 1). This test revealed one gene (*HOGA1*) at exome-wide significance (*p* < 2.7 × 10^–6^) with BMI (Table 1). The results of the skat-O test for the entire 18,379 loci reported in gene-based association analysis are shown in Supplementary Table 1.

**TABLE 1:** Top three genes associated with BMI identified by gene-based test (*p*-value < 5 × 10^–5^).

Gene	Position (GRCh37)	No. Of variants/gene set	*p*-value	Position of variant in gene set (Ma/Mi)	rsID	Variants (minor allele counts)	MAF (this study)	MAF (EAS)	MAF (EUR)	MAF (AFR)
*HOGA1*	chr10:99,344,508–99371274	6	7.07E-07	10:99,344,508 (G/T)	rs1276007639	p.Arg16Ser(1)	0.00055	na	na	na
				10:99,359,522 (C/T)	rs115282699	p.Thr185Met (11)	0.00600	0.00450	0.00017	0.00000
				10:99,359,570 (A/T)	rs1187782084	p.Asp201Val(1)	0.00055	na	na	na
				10:99,361,748 (G/T)	rs749315029	Splice Donor Variant (1)	0.00055	0.00060	0.00000	0.00000
				10:99,371,273 (C/T)	rs764224799	p.Arg281Trp (2)	0.00109	0.00000	0.00001	0.00000
				10:99,371,274 (G/A)	rs754049561	p.Arg281Gln (2)	0.00109	0.00000	0.00001	0.00000
*ZIC5*	chr13:100,617,663–100623,754	5	3.31E-05	13:100,617,663 (C/T)	rs771298495	p.Gly654Arg (2)	0.00109	0.00000	0.00000	0.00005
				13:100,617,711 (G/C)	rs761843611	p.Pro638Ala (1)	0.00055	na	0.00000	0.00000
				13:100,617,741 (C/T)	rs201567196	p.Ala628Thr (3)	0.00164	0.00030	0.00000	0.00000
				13:100,622,663 (G/A)	rs771701749	p.Pro423Ser(1)	0.00056	0.00000	0.00013	0.00000
				13:100,623,754 (A/G)	rs752163667	p.Leu59Pro(3)	0.00165	0.00320	0.00000	0.00000
*RBPMS2*	chr15:65,041,622–65041622	1	3.73E-05	15:65,041,622 (A/C)	rs6494493	intron variant (1758)	0.04144	0.03710	0.11634	0.58238

Chromosomal position is based on NCBI genome build 37/hg19. Minor allele frequency of each variant is available from the gnomAD database (https://gnomad.broadinstitute.org/). MAF, minor allele frequency; Ma, Major allele; Mi, Minor allele; EAS, east asian; EUR, european; AFR, african; na, not available.

### *HOGA1* expression in human adipocytes

**FIGURE 2:**
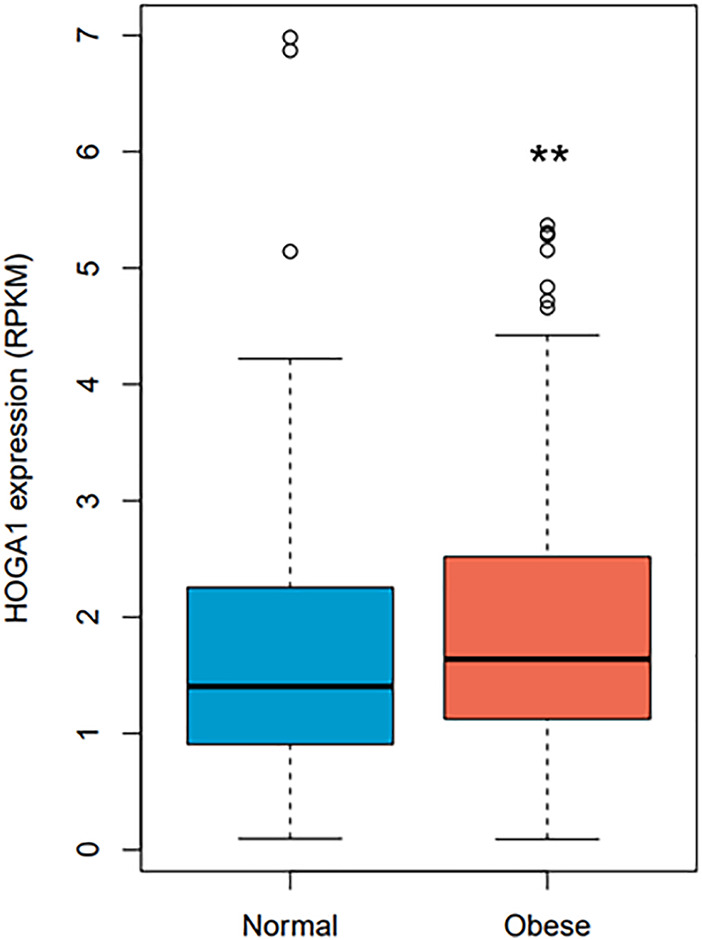
*HOGA1* expression levels in adipocytes detected from the GTEx dataset. *HOGA1* expression was compared between normal and obese individuals using RNA sequencing data of adipocyte samples. Group differences were assessed by the Wilcoxon rank-sum test (*p < 0.05, **p < 0.01, ***p < 0.001 vs normal).

*HOGA1* expression was approximately 1.2-fold higher in samples from individuals with obesity compared with from normal individuals (Wilcoxon rank-sum test *p*-value = 0.0058) (Figure 2) using RNA sequencing data of 848 adipocyte tissues from The Genotype-Tissue Expression (GTEx) portal (https://gtexportal.org/home/) (GTEx_Consortium, 2017). Linear regression analysis adjusted for age, sex, and race further revealed the significant association between BMI and *HOGA1* expression in 848 adipocyte samples (*p*-value = 0.001, *β* = 0.43 ± 0.13).

### mRNA expression of *Hoga1* in mouse 3T3-L1 cells

**FIGURE 3:**
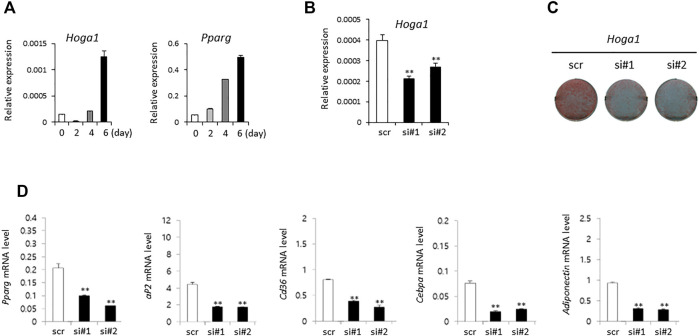
The effect of *Hoga1* knockdown on adipogenesis in mouse 3T3-L1 cells. **(A)** Relative expression levels of *Hoga1* and *Pparg* during adipocyte differentiation. **(B)** *Hoga1* knockdown efficiency of two independent siRNAs (si#1 and si#2) compared to scrambled control siRNA (scr). **(C)** Lipid accumulation during adipocyte differentiation in 3T3-L1 pre-adipocytes transfected with two independent *Hoga1*-targeting siRNAs or scr siRNA. Red dots indicate lipid droplets stained with Oil-Red-O. **(D)** Expression levels of *Pparg*, *aP2*, *Cd36*, *Cebpa*, and *Adiponectin* genes related to adipogenesis in 3T3-L1 pre-adipocytes transfected with two independent *Hoga1*-targeting siRNAs or scr siRNA. Notes: The mRNA level of each gene was measured on day 1 after treatment with each *Hoga1*-siRNA or scr siRNA by RT-qPCR analysis. Results are expressed as the mean ± SD of three independent experiments (*p < 0.05 and **p < 0.01 vs scrambled control).

The expression level of the mouse homolog of human *HOGA1* (*Hoga1*) was first examined during adipocyte differentiation of 3T3-L1 cells derived from mouse 3T3 cells. Although 3T3-L1 cells have a fibroblast-like morphology, the cells display an adipocyte-like phenotype under appropriate differentiation conditions (Green and Kehinde, 1975; Zebisch et al., 2012). Increased *Hoga1* expression was observed in response to adipogenic stimulation at the late stage together with the induced gene expression pattern of peroxisome proliferator-activated receptor gamma (*Pparg*), a key regulator of adipocyte differentiation (Figure 3A). This demonstrates that *Hoga1* is likely involved in adipogenesis.

### Effect of *Hoga1* down-regulation on adipogenesis in 3T3-L1 cells

SiRNA treatment of *Hoga1* in 3T3-L1 cells reduced *Hoga1* mRNA by 25–50%, as observed by quantitative real time polymerase chain reaction (qRT-PCR, Figure 3B). Lipid accumulation indicating adipogenic differentiation was inhibited by reduced *Hoga1* mRNA expression determined by Oil-Red-O staining (Figure 3C). Furthermore, *Hoga1* knockdown significantly reduced the mRNA expression of genes involved in adipogenesis including *Pparg*, *aP2*, *Cd36*, *Cebpa*, and *Adiponectin* (Figure 3D). These results indicate the functional role of *Hoga1* to adipocyte differentiation, and strongly imply that this gene has functional roles in adipogenesis.

### Effect of *Hoga1* down-regulation on diet-induced obese mice

**FIGURE 4:**
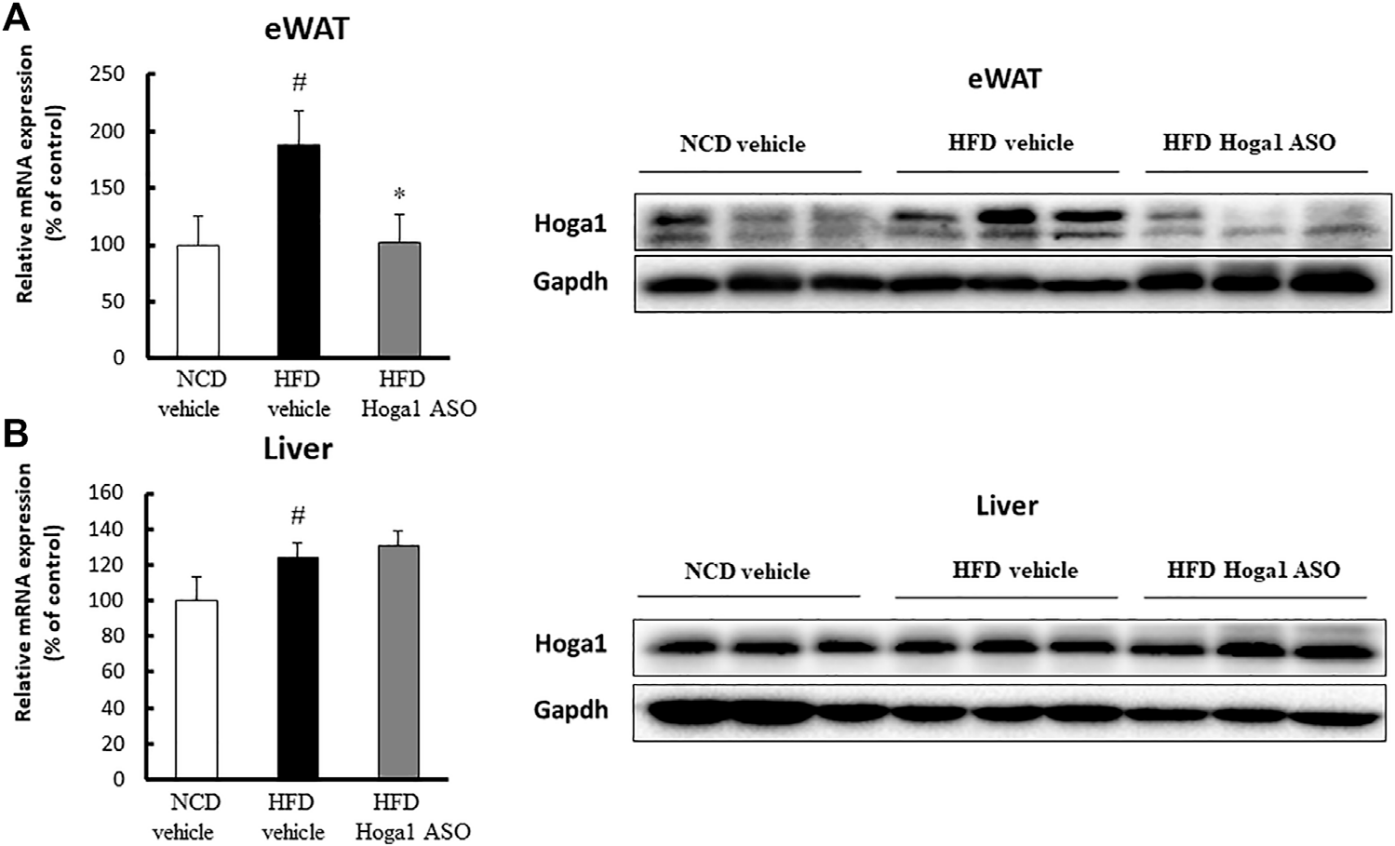
Increased *Hoga1* expression in adipose tissues of diet-induced obese mice. High-fat diet (HFD) C57BL/6J mice were treated with *Hoga1* ASO (HFD Hoga1 ASO) or scrambled control ASO (HFD vehicle) twice per week (25 mg per kg body weight per dose) for 6 weeks. In addition, normocaloric diet (NCD) mice were treated with scrambled control ASO (NCD vehicle). The mRNA and protein levels of *Hoga1* in epididymal white adipose tissue (eWAT) **(A)** and in the liver **(B)** of the NCD vehicle, HFD vehicle, and HFD Hoga1 ASO groups were measured by qRT-PCR (left panel) and Western blot (right panel) analyses, respectively. Five mice were used per group (^#^ *p* < 0.05 indicates a significant difference between the NCD vehicle and HFD vehicle; *p < 0.05 indicates a significant difference between the HFD vehicle and HFD Hoga1 ASO).

C57BL/6J mice induced with a high-fat diet (HFD) were randomly divided and intraperitoneally injected with a control oligonucleotide (vehicle) or with *Hoga1* ASO (25 mg/kg) twice a week. After 9 weeks of treatment, *Hoga1* mRNA and protein levels in the epididymal white adipose tissue (eWAT) increased compared to those in the normocaloric diet (NCD) mice. This increase in *Hoga1* mRNA and protein levels in the eWAT dropped to that of NCD mice when HFD-induced mice were treated with *Hoga1* ASO (Figure 4A). However, the significant increase of *Hoga1* was not detected in the liver of HFD-induced mice (Figure 4B). Similarly, no significant increase of this gene was observed in the liver of obese individuals in the GTEx dataset (Wilcoxon rank-sum test *p*-value = 0.297) (Supplementary Figure S1). These results strongly imply that *Hoga1* is associated with adipogenesis.

#### *Hoga1* down-regulation induces reversible weight loss and plasma lipid levels in obese mice

**FIGURE 5:**
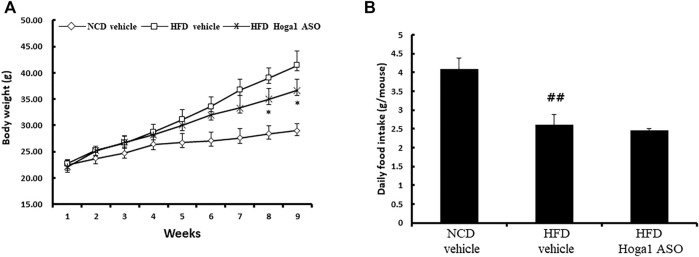
Reversible weight loss in diet-induced obese mice due to *Hoga1* knockdown. Body weight gain **(A)** and food intake **(B)** in NCD and HFD mice treated with scrambled control ASO or *Hoga1* ASO were measured from six mice per group (^##^ *p* < 0.01 indicates a significant difference between the NCD vehicle and HFD vehicle; *p < 0.05 indicates a significant difference between the HFD vehicle and HFD Hoga1 ASO).

**FIGURE 6:**
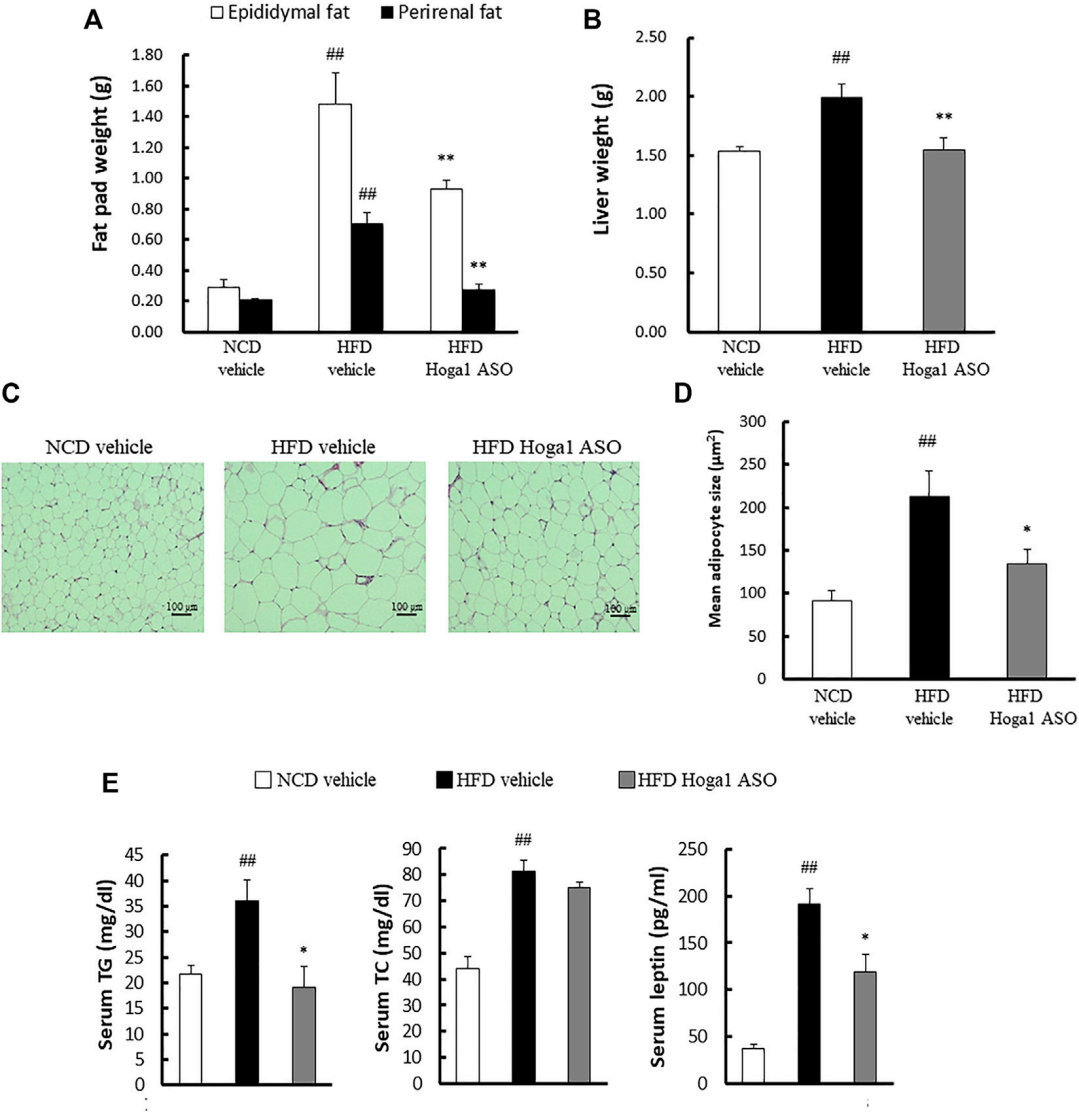
The effect of *Hoga1* knockdown on lipid properties in obese mice. Fat pad **(A)** and liver **(B)** masses of C57BL/6J mice from NCD or HFD mice treated with scrambled control ASO or *Hoga1* ASO were measured from six mice per group. **(C)** Representative images of H&E-stained sections from eWAT of NCD or HFD mice treated with scrambled control ASO or *Hoga1* ASO (×200 magnification). **(D)** Average adipocyte size per microscopic field was compared among the NCD vehicle, HFD vehicle, and HFD Hoga1 ASO groups (N = 6 per group). **(E)** Triglyceride, total cholesterol, and leptin levels of NCD and HFD mice treated with scrambled control ASO or *Hoga1* ASO were measured from six mice per group. Results are expressed as the mean ± SD of three independent experiments (^##^ *p* < 0.01 indicates a significant difference between the NCD vehicle and HFD vehicle; *p < 0.05 and **p < 0.01 indicate significant differences between the HFD vehicle and HFD Hoga1 ASO, respectively).

Treatment of mice with *Hoga1* ASO (25 mg/kg) suppressed the HFD-induced weight gain (Figure 5A). There was no effect of *Hoga1* ASO on mouse food intake during the study (Figure 5B). *Hoga1* suppression by ASO treatment in HFD-induced mice not only reduced the overall mass of epididymal fat, perirenal fat, and liver (Figures 6A,B) but also reduced adipocyte size in eWAT (Figures 6C,D). *Hoga1* ASO-treated obese mice showed decreased serum triglyceride (TG) (47%), and leptin (38%), whereas total cholesterol (TC) in the serum did not significantly change (Figure 6E).

#### *Hoga1* down-regulation inhibits insulin resistance in obese mice

**FIGURE 7:**
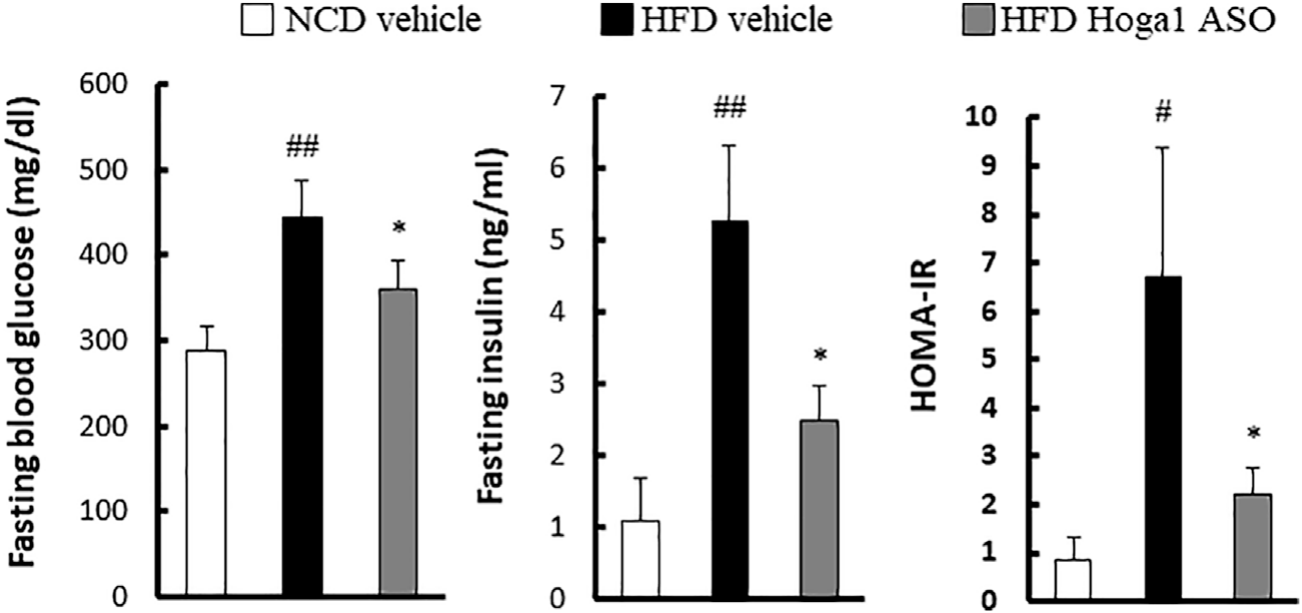
The effect of *Hoga1* knockdown on clinical indexes of insulin resistance in obese mice. Fasting blood glucose, serum insulin concentration, and HOMA-IR were compared among the NCD vehicle, HFD vehicle, and HFD Hoga1 ASO groups (N = 6 per group). Results are expressed as the mean ± SD of three independent experiments (^#^ *p* < 0.05 and ^##^ *p* < 0.01 indicate significant differences between the NCD vehicle and HFD vehicle, respectively; *p < 0.05 indicates a significant difference between the HFD vehicle and HFD Hoga1 ASO).

Obesity-induced insulin resistance has been reported (McArdle et al., 2013; Ye, 2013; Benomar and Taouis, 2019); therefore, T2D parameters were assessed in HFD-induced mice treated with *Hoga1* ASO. *Hoga1* ASO treatment ameliorated the clinical indexes of insulin resistance including glucose, insulin, and homeostatic model assessment of insulin resistance (HOMA-IR) levels in HFD-induced mice (Figure 7). This suggests that *Hoga1* down-regulation improves insulin sensitivity in obese animals.

#### *Hoga1* down-regulation ameliorates adipose tissue inflammation in obese mice

**FIGURE 8:**
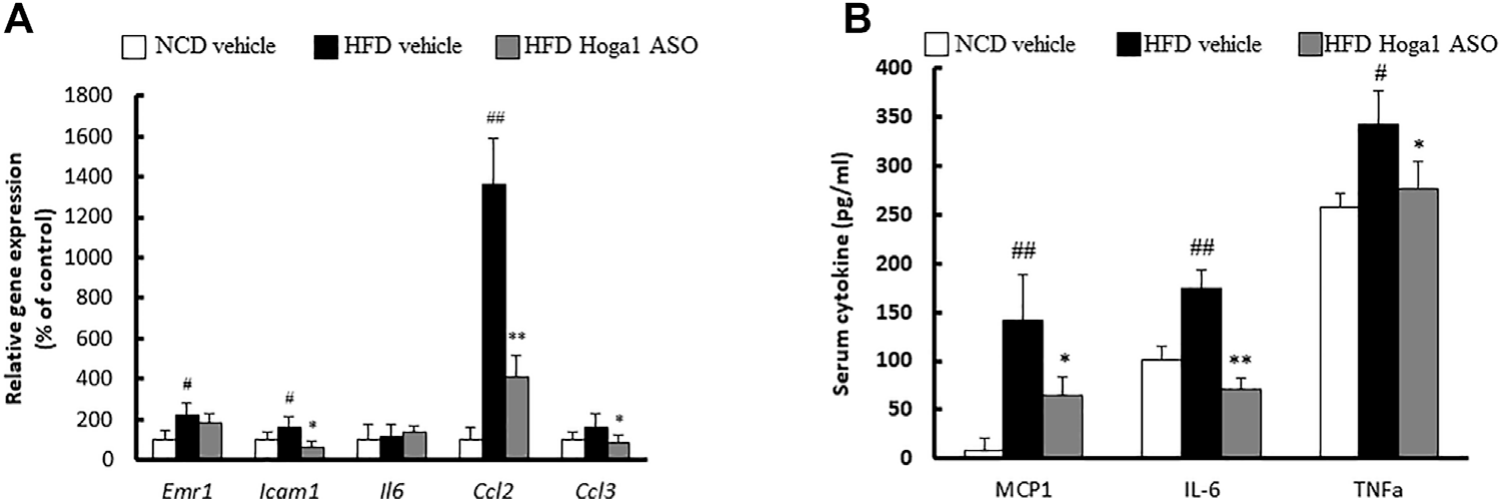
The effect of *Hoga1* knockdown on adipose tissue inflammation in obese mice. **(A)** mRNA expression of inflammatory genes in eWAT was measured from NCD and HFD mice treated with scrambled control ASO or *Hoga1* ASO (N = 5 per group). **(B)** Serum inflammatory cytokine concentrations compared among the NCD vehicle, HFD vehicle, and HFD Hoga1 ASO groups (N = 5 per group). Results are expressed as the mean ± SD of three independent experiments (^#^ *p* < 0.05 and ^##^ *p* < 0.01 indicate significant differences between the NCD vehicle and HFD vehicle; *p < 0.05 and **p < 0.01 indicate significant differences between the HFD vehicle and HFD Hoga1 ASO).

Obesity induces chronic inflammation in adipose tissues (McArdle et al., 2013). We tested whether *Hoga1* down-regulation prevents the infiltration of inflammatory macrophages into eWAT. *Hoga1* ASO treatment in HFD-induced mice significantly reduced the mRNA levels of key inflammatory genes including *Emr1*, *Icam1*, *Ccl2*, and *Ccl3* in eWAT (Figure 8A). Serum concentrations of inflammatory cytokines, including monocyte chemoattractant protein 1 (MCP-1), tumor necrosis factor-alpha (TNF-α), and interleukin-6 (IL-6) were lower in HFD-induced mice treated with *Hoga1* ASO (HFD Hoga1 ASO) than in HFD-induced mice treated with scrambled control ASO (HFD vehicle) (Figure 8B). This suggests that *Hoga1* down-regulation ameliorates adipose tissue inflammation in obese mice.

#### Molecular mechanism of *Hoga1* in adipocyte differentiation

**FIGURE 9:**
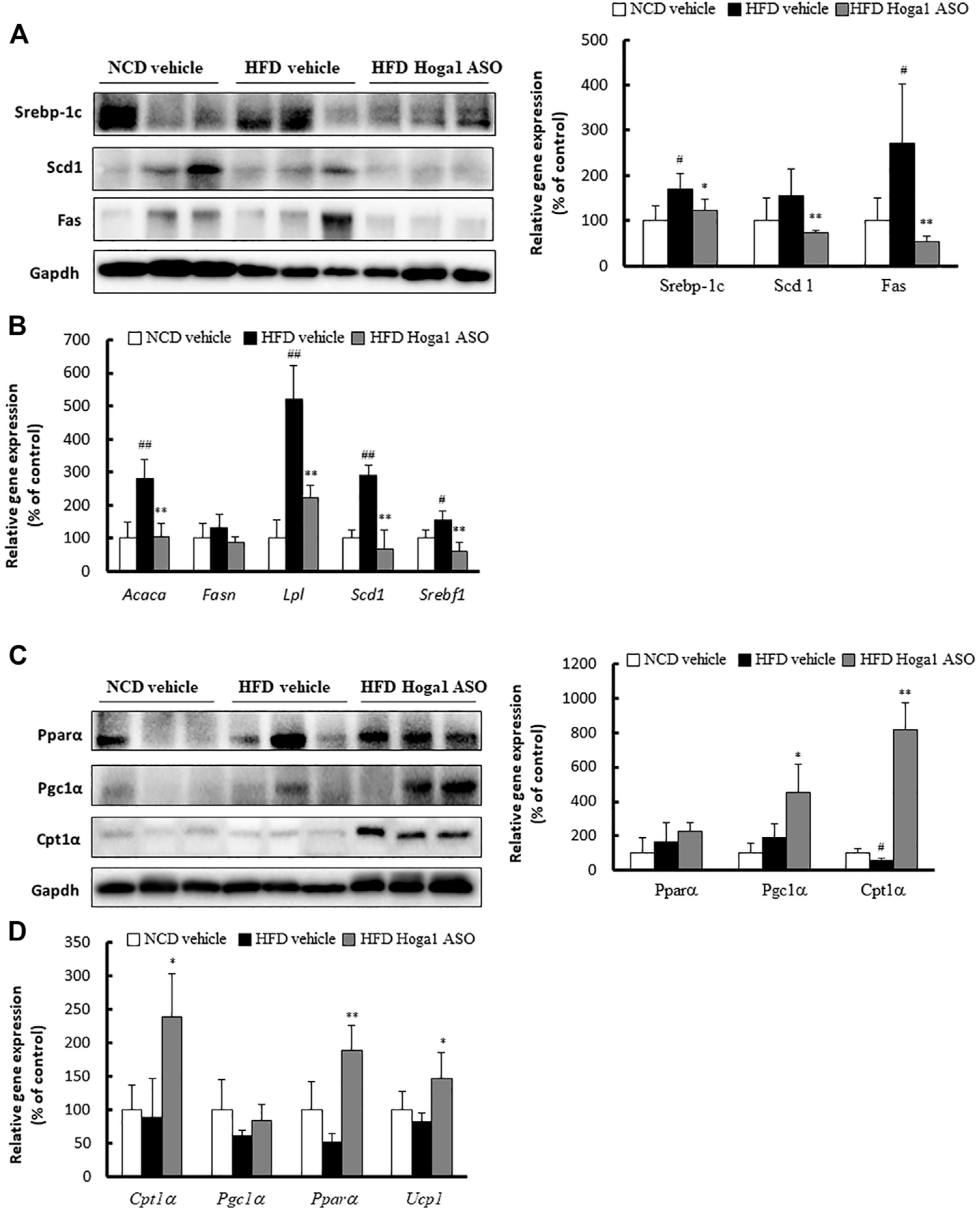
Lipogenic and lipolytic gene expression in mouse eWAT. The levels of proteins **(A)** and mRNAs **(B)** of lipogenic genes were compared in eWAT from NCD and HFD mice treated with scrambled control ASO or *Hoga1* ASO. The levels of proteins **(C)** and mRNAs **(D)** of lipolytic genes were compared in eWAT from NCD and HFD mice treated with scrambled control ASO or *Hoga1* ASO. Notes: N = 5 per group. Results are expressed as the mean ± SD of three independent experiments (^#^ *p* < 0.05 and ^##^ *p* < 0.01 indicate significant differences between the NCD vehicle and HFD vehicle; *p < 0.05 and **p < 0.01 indicate significant differences between the HFD vehicle and HFD Hoga1 ASO).

The expression levels of genes involved in lipid metabolism in eWAT were measured to gain insights into the molecular mechanism of *Hoga1* in adipogenesis. The levels of proteins (sterol regulatory element-binding protein 1c [Srebp-1c], stearoyl-CoA desaturase [Scd], and fatty acid synthase [Fas]) (Figure 9A) and mRNAs (*Acaca*, *Lpl*, *Scd1*, and *Srebf1*) (Figure 9B) involved in lipogenesis were reduced in the Hoga1 ASO-treated group. In addition, the protein (Figure 9C) and mRNA (Figure 9D) levels of lipolytic genes such as peroxisome proliferator-activated receptor alpha (*Ppara*), carnitine palmitoyl transferase 1 (*Cpt1a*), Ppar-gamma coactivator 1 alpha (*Pgc1*α), and uncoupling 1 (*Ucp1*) were increased in the Hoga1 ASO-treated group. These results indicate that *Hoga1* may enhance and suppress the expression of lipogenic and lipolytic genes, respectively.

## Discussion

In this study, exome-sequencing was employed to discover causal genes for obesity. A large portion of SNVs called from exome sequencing were rare variants. Single-variant testing using rare variants has some limitations in most association analyses because of the low power due to fewer variations and a high false positive rate due to sparse data distribution, unstable/biased parameter estimation, and inflated *p*-values (Kang et al., 2013; Lee et al., 2014). To avoid these limitations, a gene-based association analysis was designed to identify genes containing multiple risk variants that are individually and weakly associated with a univariate trait (Chung et al., 2019; Cirulli et al., 2020). The SKAT-O test used in this study includes sequence kernel association test (SKAT) and burden tests. This method was developed to maximize power in the presence of protective and deleterious variants, null variants, and a large number of causal variants with the same direction in a region (Lee et al., 2012).

The SKAT-O test for gene-based association analysis detected one gene (*HOGA1*), with exome-wide significance (*p* = 7.07 × 10^–7^) for BMI (Table 1 and Figure 1). This gene encodes mitochondrial 4-hydroxy-2-oxoglutarate aldolase 1 (HOGA1) which catalyzes the conversion of 4-hydroxy-2-oxoglutarate into glyoxylate and pyruvate (Riedel et al., 2011). Glyoxylate is then converted to oxalate by lactate dehydrogenase. HOGA1 also has a catalytic activity to convert oxaloacetate to pyruvate and carbon dioxide (Dekker and Kitson, 1992). Although the biological significance of this associated oxaloacetate decarboxylase activity is largely unknown, it suggests that the mitochondrial localization of HOGA1 is involved in the Krebs cycle. Indeed, mitochondria play a critical role in adipose tissue by regulating lipid and glucose homeostasis, energy expenditure, and adipocyte differentiation (Heinonen et al., 2015). *HOGA1* is expressed primarily in the liver and kidney, and genetic variation of this gene is observed in patients with primary hyperoxalurea type III (Fang et al., 2019; Huang et al., 2019). This is the first study identifying the association of *HOGA1* with BMI. In the GTEx dataset, a significantly increased expression of *HOGA1* was observed in adipocytes from obese people (Figure 2), suggesting that *HOGA1* is biologically relevant to adipogenesis.

We validated the functional role of *Hoga1* in adipogenesis using mouse 3T3-L1 cells that exhibit a fibroblast-like morphology but have an adipocyte-like phenotype under appropriate differentiation conditions (Green and Kehinde, 1975; Zebisch et al., 2012). *Hoga1* mRNA expression increased in mouse 3T3-L1 adipocytes during adipogenesis. In addition, adipogenesis was markedly inhibited in *Hoga1* siRNA-treated 3T3-L1 cells. Based on these findings, we hypothesize that *Hoga1* might act as an endogenous factor regulating adipocyte function.

Hormones regulate adipocyte differentiation via transcription factors and target genes involved in adipogenesis, including PPARγ, C/EBPα, aP2, CD36, and adiponectin (Lee et al., 2018). C/EBPβ is a key transcription factor involved in early adipocyte differentiation by promoting PPARγ expression which activates C/EBPα expression. Subsequently, PPARγ and C/EBPα activate the expression of numerous genes involved in terminal adipocyte differentiation (Tontonoz et al., 1994). Our study found that *Hoga1* siRNA treatment decreased the expression of Pparγ, C/ebpα, aP2, Cd36, and adiponectin, indicating that *Hoga1* knockdown suppressed intracellular lipid accumulation through the Pparγ and C/ebpα pathways.

The anti-obesity effect of *Hoga1*-targeted ASO was corroborated and extended in diet-induced obese mice. We hypothesized that the intraperitoneal route of injection with *Hoga1* ASO contributed to adipose tissue infiltration. Treatment with *Hoga1* ASO resulted in (i) reduced *Hoga1* expression in eWAT, (ii) decreased adipocyte size and the expression of lipogenic key factors in the eWAT, and (iii) decreased inflammatory cytokine levels that are highly associated with metabolic dysregulation in tissue and serum. Weight loss in obesity ameliorates inflammatory factors (Forsythe et al., 2008); however, rapid and sustained weight loss is rarely possible. Mice with deletion of genes that play an important role in obesity-induced inflammation had improved insulin resistance and reduced hepatic lipid accumulation and liver weight compared to mice maintained on a HFD despite negligible changes in body weight (Kanda et al., 2006; Wueest et al., 2010). Our study showed a small but significant weight loss at the end of the 7-weeks observation period after *Hoga* ASO treatment, suggesting that prolonged treatment may lead to additional weight loss. A more intriguing finding was that the knockdown of *Hoga1* expression drastically reduced adipose tissue size, adipose tissue weight to body weight ratio, and adipocyte diameter in epididymal fat depots.

Dysregulation of lipid metabolism is one of the major pathogenesis of obesity (Savage et al., 2007; Smilowitz et al., 2009). Circulating free fatty acids (FFAs) in the plasma are absorbed by metabolic organs such as adipose, liver, and muscle tissues and are converted to TGs via lipogenesis and lipid oxidation pathways (Olefsky, 2008). Lipogenic enzymes, including FAS and SCD, synthesized in hepatocytes and adipocytes, are regulated by SREBP-1c, a key transcription factor inducing TG biosynthesis (Harvatine and Bauman, 2006). Increases in these lipogenic factors significantly increase the levels of FFAs and cholesterol, leading to insulin resistance. However, lipolytic factors such as PPARα, CPT-1, and PGC-1α activate fatty acid oxidation and clearance. Thus, reduced lipogenic proteins and increased lipolytic proteins ameliorate obesity and dyslipidemia in HFD-induced obese mouse models (Boden, 2006). In this context, our study demonstrated that *Hoga1* may enhance and suppress the expression of lipogenic and lipolytic genes, respectively in HFD-induced obese mice during adipogenesis. HOGA1-lipogenic (or lipolytic) protein interactions need to be elucidated to understand the molecular mechanism exerted by *HOGA1* in adipogenesis.

Modulating the proliferation or differentiation of adipocytes may yield promising drug targets (Stumvoll et al., 2005; Biddinger and Kahn, 2006). However, our understanding of the complex genetic networks underlying adipocyte differentiation is incomplete, and little is known regarding which proteins are responsible for the binding and localization of transcriptional complexes in adipocytes. This study was conducted to leverage findings from the genetic association analysis to provide a biological understanding of obesity for future drug development. Overall, our data indicate that HOGA1 may act as an important regulator of adipogenesis. Therefore, targeting HOGA1 could be a potential therapeutic target for obesity.

## Data Availability

The original contributions presented in the study are included in the article/Supplementary Material, further inquiries can be directed to the corresponding authors. Exome sequencing data are available from the Clinical & Omics Data Archive (CODA) in the Korea National Institute of Health (https://coda.nih.go.kr) with the CODA accession number R001814 upon reasonable request.
