# Supplementary figures and images for: Genetic association-based functional analysis detects HOGA1 as a potential gene involved in fat accumulation
Source: Front Genet. 2022 Aug 12;13:951025. doi: 10.3389/fgene.2022.951025 (PMC9412052; doi:10.3389/fgene.2022.951025)

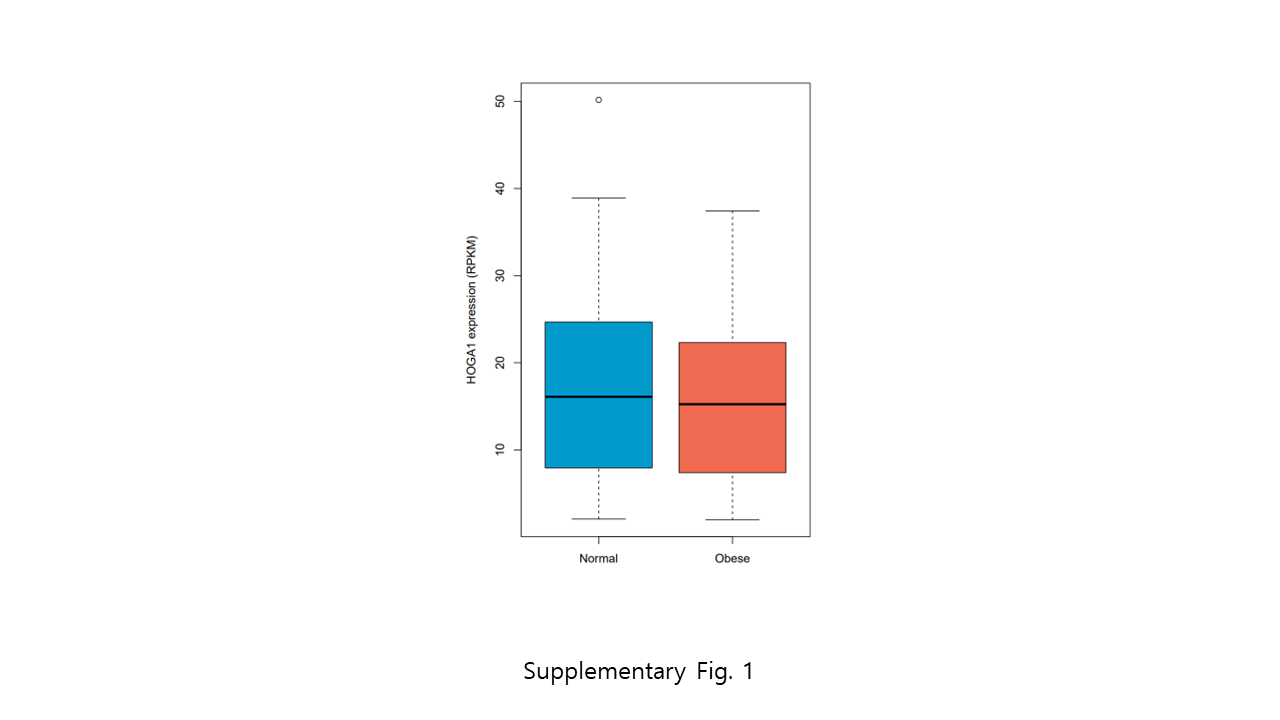

Supplement: Supplementary file 3 [file Image1.TIF]
